# Be Quiet! Effects of Competing Speakers and Individual Characteristics on Listening Comprehension for Primary School Students

**DOI:** 10.3390/ijerph20064822

**Published:** 2023-03-09

**Authors:** Chiara Visentin, Matteo Pellegatti, Maria Garraffa, Alberto Di Domenico, Nicola Prodi

**Affiliations:** 1 Department of Engineering, University of Ferrara, Via Saragat 1, 44122 Ferrara, Italy; matteo.pellegatti@unife.it (M.P.); nicola.prodi@unife.it (N.P.); 2 Institute for Renewable Energy, Eurac Research, A. Volta Straße/Via A. Volta 13/A, 39100 Bolzano-Bozen, Italy; 3 School of Health Sciences, University of East Anglia, Norwich Research Park, Norwich NR4 7TJ, UK; m.garraffa@uea.ac.uk; 4 Department of Psychological, Health and Territorial Sciences, University of Chieti-Pescara, Via dei Vestini 31, 66100 Chieti, Italy; alberto.didomenico@unich.it

**Keywords:** classroom acoustics, noise, children, listening comprehension, cognitive abilities, noise sensitivity, attention, working memory

## Abstract

Students learn in noisy classrooms, where the main sources of noise are their own voices. In this sound environment, students are not equally at risk from background noise interference during lessons, due to the moderation effect of the individual characteristics on the listening conditions. This study investigates the effect of the number of competing speakers on listening comprehension and whether this is modulated by selective attention skills, working memory, and noise sensitivity. Seventy-one primary school students aged 10 to 13 years completed a sentence comprehension task in three listening conditions: quiet, two competing speakers, and four competing speakers. Outcome measures were accuracy, listening effort (response times and self-reported), motivation, and confidence in completing the task. Individual characteristics were assessed in quiet. Results showed that the number of competing speakers has no direct effects on the task, whilst the individual characteristics were found to moderate the effect of the listening conditions. Selective attention moderated the effects on accuracy and response times, working memory on motivation, and noise sensitivity on both perceived effort and confidence. Students with low cognitive abilities and high noise sensitivity were found to be particularly at risk in the condition with two competing speakers.

## 1. Introduction

Concentrating on the task at hand is hard when people are talking in the background. This is true for an adult working in the office, and even more so for a primary school child listening to the teacher’s voice among the chatter of her/his peers. 

The ability to segregate the to-be-attended speech stream (the teacher’s voice) from the global auditory scene (students’ chatter, sounds coming from outside, technological equipment noise) relies on both the peripheral auditory system and central auditory and cognitive processing. Both systems develop with age, with complete maturation only taking place during late childhood (>10 years) [1,2]. As a consequence, children will be less proficient than adults at perceiving speech-in-noise; even when adequate peripheral encoding is guaranteed, there might be the possibility that competing sounds disrupt higher-order processing. Difficulties in speech perception further increase when the background noise is composed of a small number of speech streams, resulting in a signal with intelligible and meaningful content. Beside energetic masking (due to the physical overlap between the two streams) this type of noise also causes informational masking with higher-level consequences (attentional capture, semantic interference, and increased cognitive load) [3].

Concerning the number of competing talkers, literature on adults indicates an advantage in speech perception in a single speech stream compared to steady-state noise (in anechoic conditions), due to the listener’s ability to exploit short periods with high signal-to-noise ratios (SNRs) and glimpse part of the target speech [4]. However, this advantage disappears as the number of competing speakers increases from one to three, thus decreasing the opportunity to glimpse and increasing informational masking. No difference from speech perception in steady-state noise was observed starting from four competing talkers [5,6]. Less information on the effect of the number of competing talkers on speech perception is available for children, even though this type of noise is common in the classroom setting, where a small number of children might be talking simultaneously to the teacher. It is well-assessed that larger child–adult differences are present for speech-in-speech (two-talkers) compared to speech-in-noise perception [7], and that the two processes follow different developmental trajectories [8]. Moreover, in comparison to adults, children show a less mature use of the binaural cues that support speech-in-noise perception, even in mild reverberation [9]. The effect of the number of competing talkers (one or four; SNR +5 dB) on children’s passage comprehension was investigated by von Lochow et al. [10,11], finding no effect of the listening condition on task accuracy or perceived effort.

A growing body of literature points toward the need to go beyond performance (i.e., accuracy, number of correct responses in a given task) when assessing the effect of the classroom sound environment on students. Indeed, even when performance is stable or at the ceiling, there might be an increase in listening effort, defined as “the deliberate allocation of resources to overcome obstacles in goal pursuit” [12]. The Framework for Understanding Effortful Listening (FUEL) [12] postulates a relationship between the cognitive demands of a listening task, that might originate from acoustic challenges at the listener, talker, or environment level, and the listener’s supply of cognitive capacity. Adverse acoustic conditions force the listeners to deploy cognitive resources to suppress the task-irrelevant stimuli, thereby experiencing an increase in listening effort and sparing fewer capacities for the processing and understanding of the content. The concept of effort is especially relevant for students learning in classrooms that are often too noisy and/or have too much reverberation [13,14]. Moreover, effortful cognitive demands sustained over time might lead to listening-related fatigue, associated with slowed information processing and a decreased level of attention [15], and eventually to communicative disengagement [16]. 

In addition to increased effort, (chronic and short-term) exposure to background noise can affect the motivational component of learned helplessness [17,18], with children persisting less when required to perform a task and giving up more easily [19]. The role of motivation in effortful listening is acknowledged by the FUEL [12]. It influences the listener’s engagement in the task and indirectly affects the deployment of cognitive resources and the speed at which processing is performed [20]. Motivation is, thus, intended as a listener’s personal state that interacts with the cognitive demands of the task in determining the perceived effort [21]. Motivation is crucial for learning, as motivated students can select and adopt strategies to persevere in and complete a task, even in challenging conditions, and control their attention better. What motivates students to listen in the classroom is still unclear, however, even though roles are suggested for the pleasantness of the teacher’s voice [22,23], reverberation time [24], and more generally unstimulating and non-arousing classroom features [25]. 

Finally, challenging acoustic conditions might also impact the listener’s confidence in her/his ability to complete a task. Confidence is a subjective measure of the awareness of knowing, indicating how much people believe they are guessing or not when making a judgment [26]. Previous studies on the effects of face masks on confidence showed that wearing a surgical mask reduced confidence in reading facial emotions [27] and in accuracy while doing a speech perception task in multi-talker noise (with a further decrease in confidence when increasing the number of talkers from one to three [28]). Confidence is related to metacognition, i.e., the degree to which the listeners are capable of monitoring whether they have understood the message correctly or not. This aspect might be especially relevant in the case of students, as adequate metacognitive monitoring of communication in the classroom would trigger coping strategies (e.g., asking the teacher to speak louder or more slowly).

Students are not equally at risk from background noise interference during lessons. It is well-assessed that children with special education needs (learning- or language-based) or hearing impairments are significantly more negatively affected by noise, compared to the other learners [29]. However, even for children with neurotypical development and normal hearing, differences in task performance and reactions to the acoustic quality of the learning space can be observed [30] due to their cognitive abilities and subjective perception [31]. Therefore, as respect for classroom acoustic standards and normative values will not ensure the well-being of every student in the classroom [32], an increasingly student-centered approach has to be established to create inclusive spaces, by understanding the individual factors that modulate the effect of the sound environment on learning.

According to the Ease of Language Understanding (ELU) model [33], listeners rely on cognitive skills such as working memory capacity and attention to understand speech in challenging conditions. These cognitive abilities moderate the effect of background noise on speech perception. For instance, children with greater working memory capacities will have better speech-in-noise performance than students with lower working memory capacities [34,35], and young children will be less proficient in speech-in-noise perception due to their still immature auditory selective attention skills [36]. Both cognitive processes are core “executive functions”, defined as “top-down mental processes needed when you have to concentrate and pay attention, when going on automatic or relying on instinct or intuition would be ill-advised, insufficient, or impossible” [37]. Working memory involves storing and making sense of information that is no longer perceptually present (e.g., making sense of a spoken sentence). Selective attention represents resistance to external distractors. It is the aspect of inhibitory control that allows selectively attending to a stimulus, while simultaneously suppressing attention to other salient stimuli (i.e., stimuli attracting attention whether we want it to or not, such as our name pronounced aloud or a sudden movement). 

The conceptual model of Reinten et al. [31] indicates that noise sensitivity might moderate the effects of noise on cognition. Individuals with high noise sensitivity are believed to have a lower perceptual threshold and might be particularly impaired by the presence of challenging acoustic conditions. Whereas a strong association between noise sensitivity and annoyance is well-documented for children in schools [38], the relation to cognition is rarely explored. For university students in open-plan environments, it was found that noise sensitivity mediates the effect of noise in a writing task [39], but not in a collaborative task [40]. For children aged 8 to 10 years, noise sensitivity was found to moderate the effect of a two-talker masker sound level on perceived effort in a comprehension task [41]. 

This study aimed to explore the relationship between individual factors and listening conditions (background noise with two or four competing speakers) on a sentence comprehension task presented to primary school students, concerning task accuracy, listening effort, motivation, and confidence. The following research questions were formulated:

(i) Does the number of competing speakers influence students’ accuracy and effort in a sentence comprehension task? Two measures of listening effort were included in the study: response time in a single-task paradigm (behavioral measure [42,43]) and self-reported effort (subjective measure).
(ii) Do the individual factors (selective attention, working memory, noise sensitivity) moderate the effect of the listening condition on students’ accuracy and effort in the task?
(iii) What are the effects of the classroom sound environment on students’ motivation and confidence in doing the task?

## 2. Materials and Methods

### 2.1. Participants

A total of 79 students from three different schools in Ferrara (Italy) participated in the experiment. Students with special education needs (n = 6) and those scoring under the chance level in the task performed in quiet (n = 2) were removed from the dataset. No students had known hearing impairments. The final sample of participants included 71 students in grades 5 and 7, aged between 10 and 13 years (grade 5: n = 24, 13 female, mean age ± sd: 10.2 ± 0.4 years; grade 7: n = 47, 20 female, 12.5 ± 0.5 years).

### 2.2. Measures

#### 2.2.1. Selective Attention

In the experiment, selective attention was tested using Simon and Flanker tasks presented in the visual domain. Both tasks were implemented and presented online using the PsyToolkit platform [44,45]. 

In the Simon task, participants were presented with two visual stimuli (two colored circles). The rule was as follows: when stimulus A (red circle) appeared they had to press on the left side of the tablet, whereas when stimulus B (green circle) appeared they had to press on the right. Only one stimulus appeared at a time; either stimulus could appear on the right or the left of the tablet. There were 150 trials (50% congruent). Each trial terminated after 4000 ms.

In the Flanker task, the children saw a row of five letters and were asked to attend to the one presented at the center (either pressing on the left or the right of the tablet, depending on the letter), ignoring the flanking stimuli surrounding it. There were 120 trials; each trial terminated after 3000 ms. 

In both tasks, slower responses were expected in the incongruent condition (Simon task: stimulus on the side opposite its associated response, Flanker task: mismatch between the response required by the central letter and the response associated with the flanking letters) compared to the congruent condition, due to the need to inhibit the automatic response. For both tasks, accuracy and response time (RT) were recorded at the trial level. Trials with RTs under 150 ms were excluded from the analysis, because the time was too short to allow the perception of the stimulus. Additionally, the Median Absolute Deviation criterion (MAD) [46] with a deviation of 2.5 units was used to detect and remove outliers. The difference between the average RTs in incompatible and compatible trials was used as a performance measure. Participants were then sorted into two groups (low/high selective attention) based on the median score of the sample for each test.

#### 2.2.2. Working Memory Capacity

Verbal working memory capacity was tested using a 2-back task. The 2-back task is a continuous recognition task in which participants have to decide whether a stimulus was previously presented or not. A continuous sequence of four different letters (A–D) was shown; for each item, children had to determine whether the current letter was identical to the stimulus presented 2 trials back. Participants had to tap on the tablet whether the response to the trial was “yes”. There were 60 trials (30% correct) that were presented in random order. 

The task was implemented and presented online using the PsyToolkit platform [44,45]. Task performance was assessed using the discrimination index *d’* [47], which is a composite index calculated from hits (i.e., participant correctly touching the device in response to a target) and false alarms (i.e., participant incorrectly touching the device in response to non-targets). The better a participant maximizes hits (i.e., minimizes misses) and minimizes false alarms (i.e., maximizes correct rejections) the better the discrimination index, and the better the participant is able to discriminate target from non-target when performing a task. Participants were then sorted into two groups (low/high working memory) based on the median score of the sample. 

#### 2.2.3. Noise Sensitivity

Noise sensitivity was assessed using a reduced Italian version of the Weinstein Noise Sensitivity Scale [48]. The children had to indicate their agreement on five statements related to their sensitivity to noise. For each statement, the level of agreement could be chosen on a 5-point scale (from 1 “not at all” to 5 “very much”). 

The questionnaire was implemented in Google Forms and presented online to the students. To derive a single score, the score of the last statement was flipped to match the direction of the others (i.e., higher scores imply a higher sensitivity to noise) and the average of the scores over the five statements was calculated. Participants were then sorted into two groups (low/high noise sensitivity) based on the median score of the sample.

#### 2.2.4. Sentence Comprehension Task

The experimental task was designed to assess the listener’s ability to comprehend a sentence in noise. Materials for the task were adapted from a standardized sentence-to-picture test developed for Italian (Comprendo) [49]. 

**Figure 1:**
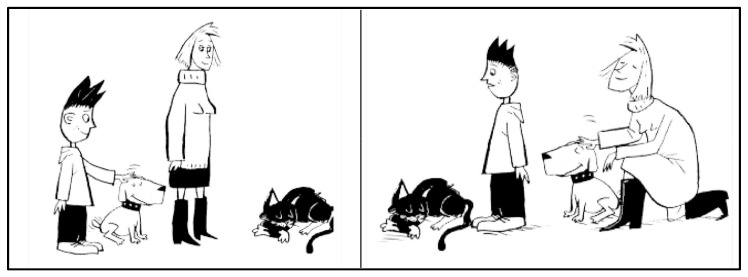
Set of two images associated with a target sentence in the comprehension task. The sentence is: “The boy looks at the cat and the mum cuddles the dog”.

For each listening condition, 15 sentences were presented to the participants via headphones. The sentences were split into three blocks, in which the sentences were counterbalanced by syntactic complexity. For each trial, participants listened to the playback of a sentence, with the background noise starting almost one second before the sentence and ending at the same time. At the audio offset, two images appeared on the tablet and participants had to select the image that best matched the sentence content (Figure 1). The task was time-limited to 15 s. Accuracy and response time (the time elapsed between the end of the audio playback and the answer selection) were recorded for each sentence.

#### 2.2.5. Subjective Assessments

Questions to elicit the self-ratings were presented to the participants at the end of each listening condition. The questions were:

(i) “How hard did you have to work to understand the previous sentences?” (subjective rating of listening effort [50]);
(ii) “How important was it to you to perform well in the task?” (subjective rating of motivation [51]);
(iii) “How confident were you about your listening experience?” (subjective rating of confidence [28]).

Participants answered the three questions using visual analog scales, ranging from 0 to 100 in increments of one. The slider was initially positioned on the midpoint of the scale. Verbal anchors (Not at all, Extremely) were positioned at each endpoint of the slider bar.

### 2.3. Listening Conditions

**Figure 2:**
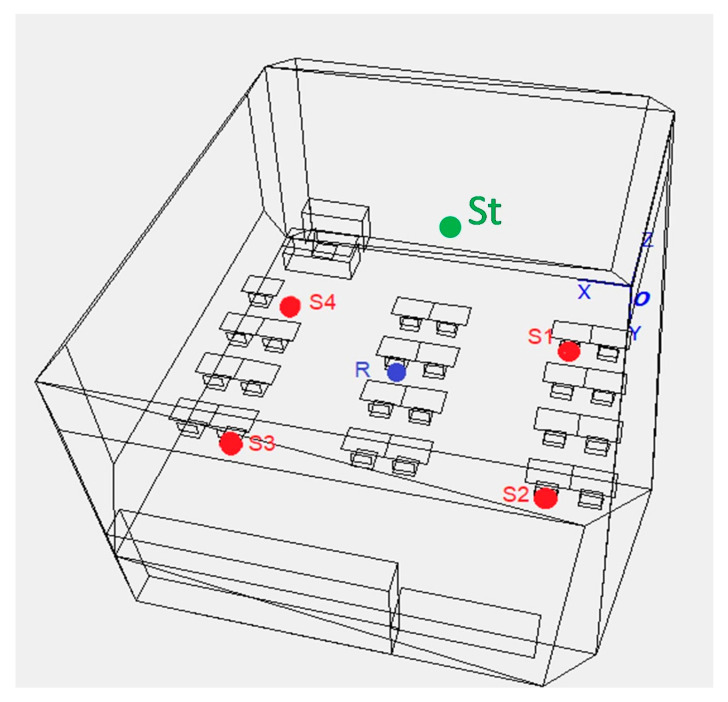
Virtual classroom with the positions of the talker (S_t_), receiver (R), and the two (S1 and S3) and four (S1–S4) competing talkers.

Participants completed the comprehension task in three listening conditions, which varied by type of background noise: quiet, two-talker noise, and four-talker noise. The conditions were created in the room acoustic modeling software ODEON v.14, by simulating a virtual classroom with a volume of 256 m^3^ and a reverberation time at the medium frequencies of 0.73 s. The reverberation time complies, also in the octave-band distribution, with the Italian acoustic standard on schools (UNI 11532-2). In the virtual classroom, the listener was positioned in the center of the area where the students usually sit, with the competing talkers surrounding it at 1.5 m of distance (Figure 2). A third speech source was simulated at the teacher position, close to the front of the classroom, in line with the receiver. The binaural impulse responses simulated in the classroom were then convolved with the anechoic recordings of four children (three female, age range: 7–11 years), reading aloud passages from different age-appropriate books, and a female speaker reading the sentences of the comprehension task. Each child’s voice originated from a single position. In the two-talker condition, only the competing talkers in positions S1 and S3 (front-right and back-left, see Figure 2) were included.

In all listening conditions the speech level was set to 60 dB(A) and the background noise level was set to 55 dB(A), to obtain an SNR of +5 dB. This SNR is representative of the acoustic conditions in actual classrooms during lessons [52] and guarantees an uncompromised speech signal audibility. In the quiet condition, no background noise was played back.

### 2.4. Procedures 

The study had a repeated-measures design, with all students performing the experimental task in the three listening conditions. The order of the conditions was counterbalanced across the students of each class. An ecological class-wise experimental paradigm was chosen; students took part in the experiment as a whole class, in the classroom in which they usually have lessons. Each class completed the experimental task in a one-hour session, during the morning school hours, and the cognitive tests and noise sensitivity self-report in a quiet, one-hour session a week later. 

The sentence comprehension task and the self-assessments were programmed using the Gorilla Experiment Builder (https://www.gorilla.sc (accessed on 20 January 2023)) [53] and completed online using tablets. Sound stimuli were delivered via headphones (Sony MDR-ZX310), whose frequency response was compensated for.

### 2.5. Data Analysis 

All data were analyzed using *R* [54] and *RStudio* [55] with the *lme4* [56] and the *afex* [57] packages. Generalized linear mixed-effect models (GLMMs) were used to analyze accuracy (coded in a binary format: 0/wrong, 1/correct) and response time data (having a positively skewed distribution). Linear mixed-effects models (LMMs) were used for the subjective assessments.

Fixed effects included in the models were listening condition (quiet, two-talkers, four-talkers), selective attention (low/high), working memory capacity (low/high), and noise sensitivity (low/high). The two-way interactions between listening conditions and individual factors were also included in the models. Random effects were included to account for variance in participants and items (sentences). As the two measures of selective attention (Simon task and Flanker task) were correlated (*r* = 0.35, *p* = 0.002), two different models were created for each outcome (including selective attention, measured either with the Simon task or the Flanker task) to avoid multicollinearity issues.

First, the model with all main effects and interactions was examined using the mixed package. Then a reduced model with only significant effects was created. In Section 3, only the reduced model is reported.

## 3. Results

### 3.1. Sentence Comprehension: Accuracy

**Figure 3:**
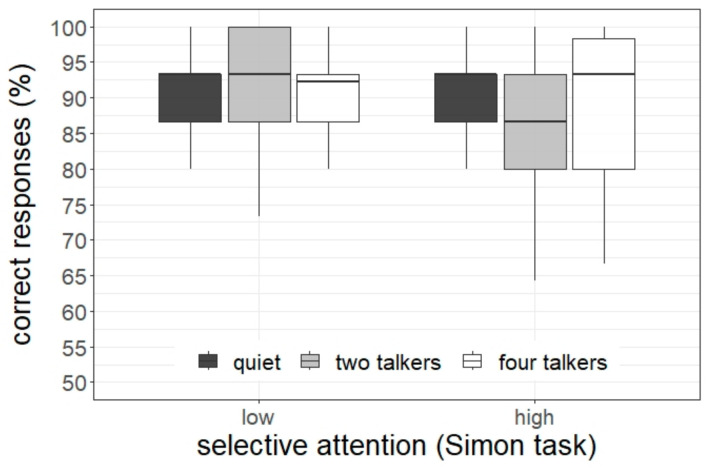
Task accuracy as a function of students’ selective attention (as assessed by the Simon task) and listening condition.

The statistical analysis of the accuracy data indicated a significant main effect of the listening condition (χ^2^[2] = 11.08, *p* = 0.004), and a significant interaction between the listening condition and the selective attention, as assessed by the Simon task (χ^2^[2] = 5.96, *p* = 0.048). No main effect of selective attention was found (*p* = 0.74). The significant interaction is depicted in Figure 3. The pairwise tests revealed that children with high selective attention had a lower task accuracy in the listening condition with two talkers compared with quiet and the four-talker condition (2T < quiet: difference = 6.3 percentage points, *z* = 3.79, *p* < 0.001; 2T < 4T: difference = 4.4 percentage points, *z* = 2.80, *p* = 0.005). In contrast, there was no difference between the listening conditions for children with low selective attention skills (all *p*s > 0.14). 

### 3.2. Sentence Comprehension: Response Time

**Figure 4:**
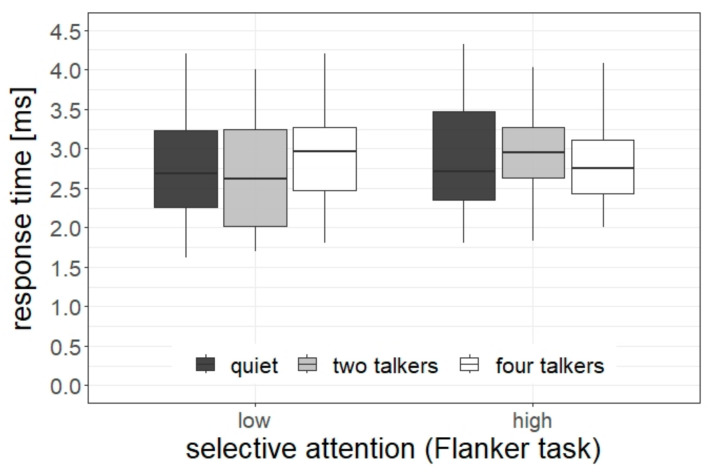
Response time (correct responses only) as a function of students’ selective attention (as assessed by the Flanker task) and listening condition.

The statistical analysis of the response time data (correct responses only) indicated a significant interaction between the listening condition and the selective attention, as assessed by the Flanker task (χ^2^[2] = 13.73, *p* = 0.001). The main effects were not significant (*p*s > 0.06). Figure 4 displays the significant interaction. The pairwise test revealed that children with low selective attention skills had longer RTs in the listening condition with four talkers compared with quiet and the two-talker condition (4T > quiet: difference = 251 ms, *z* = 2.14, *p* = 0.03; 4T > 2T: difference = 221 ms, *z* = 3.13, *p* = 0.002). In contrast, children with high selective attention had longer RTs in the listening condition with two talkers compared to quiet (2T > quiet: difference = 223 ms, *z* = 3.01, *p* = 0.003).

### 3.3. Subjective Ratings of Effort

**Figure 5:**
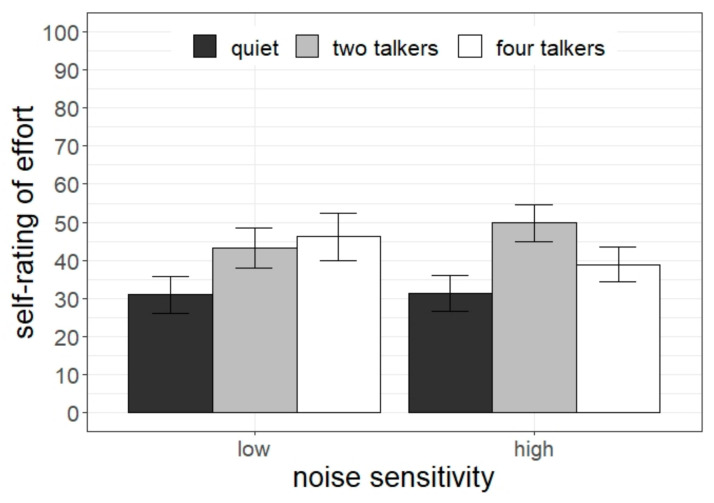
Subjective assessment of listening effort as a function of students’ noise sensitivity and listening condition. Error bars indicate standard errors.

The statistical analysis of the self-reported listening effort indicated a significant main effect of the listening condition (χ^2^[2] = 12.56, *p* = 0.02), and a significant interaction between the listening condition and the self-rated noise sensitivity (χ^2^[2] = 6.83, *p* = 0.033). The interaction is depicted in Figure 5. The pairwise test revealed that children with low noise sensitivity perceived more effort in the four-talker condition compared with quiet (4T > quiet: difference = 16.2, *t* = 2.29, *p* = 0.02). Students with high noise sensitivity perceived more effort in the two-talker condition compared with quiet and the four-talker condition (2T > quiet: difference = 20.0, *t* = 3.45, *p* < 0.001; 2T > 4T: difference = 14.2, *t* = 2.42, *p* = 0.017).

### 3.4. Subjective Ratings of Motivation

**Figure 6:**
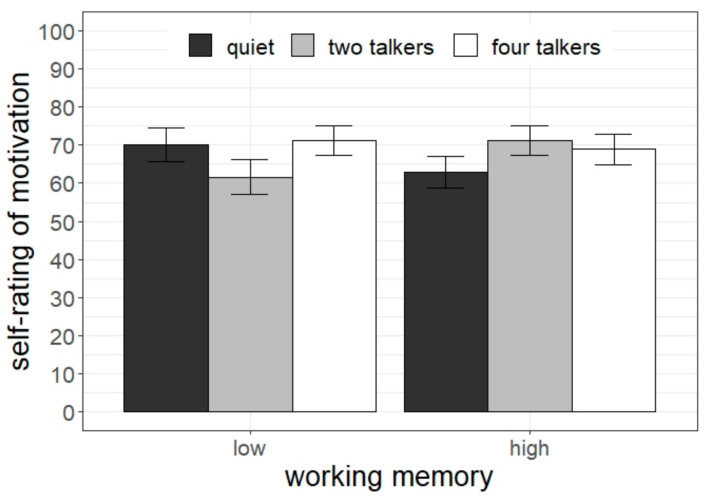
Subjective assessment of motivation as a function of students’ working memory and listening condition. Error bars indicate standard errors.

Ratings of motivation are displayed in Figure 6. Analysis revealed a significant interaction between listening condition and working memory capacity (χ^2^[2] = 17.20, *p* < 0.001). Pairwise comparisons revealed that children with low working memory capacity were less motivated in doing the task in the two-talker condition compared to the other two conditions (2T < quiet: difference = 9.55, *t* = 2.59, *p* = 0.011; 2T < 4T: difference = 10.34, *t* = 2.77, *p* = 0.006). Differently, children with high working memory capacity were more motivated in doing the task in the two-talker noise than in quiet (2T > quiet: difference = 10.86, *t* = 3.00, *p* = 0.003).

### 3.5. Subjective Ratings of Confidence

**Figure 7:**
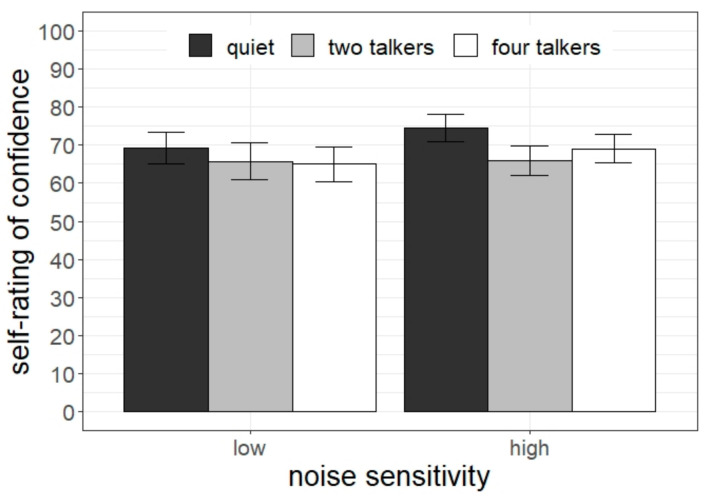
Subjective assessment of confidence as a function of students’ noise sensitivity and listening condition. Error bars indicate standard errors.

Ratings of confidence in doing the task are displayed in Figure 7. Analysis revealed a significant interaction between listening condition and noise sensitivity (χ^2^[2] = 6.18, *p* = 0.045). Pairwise comparisons revealed that children with low noise sensitivity perceived no difference between the listening conditions (*p*s > 0.12), whereas children with high noise sensitivity were less confident in doing the task in the two-talker condition than in quiet (2T < quiet: difference = 10.3, *t* = 2.81, *p* = 0.006).

## 4. Discussion

In this work, primary school children were presented with a listening comprehension task in a multi-talker background noise. An ecological setting was used for the experiment (virtualized, complex acoustic scenes presented in real classrooms, with all the students present) to elicit behavioral responses from the students that were as similar as possible to those experienced during learning. 

Concerning the first research question (effect of the number of competing speakers), results showed that the listening condition had a small direct effect on listening comprehension (accuracy and response time). Accuracy, in particular, was close to the ceiling both in quiet and in noise, indicating that the task was well within reach of the students. Differently from accuracy and response time, a significant main effect of the listening conditions was found on the perceived effort. In line with the frameworks of listening effort (ELU and FUEL), children perceived a greater effort in the multi-talker conditions compared with quiet. This supports the idea that listening in the presence of noise calls for the deployment of additional cognitive resources to maintain task accuracy, which is experienced by the children as an increase in perceived effort. Our result aligns closely with the findings in [10,11], showing no effects of increasing the number of competing speakers (one to four, SNR + 5dB) on the accuracy or perceived effort of a passage comprehension task, for 7- to 12-year-old children. It seems that a difference might exist between children and adults, regarding the effect of the number of competing speakers, with adults experiencing a decrease in sentence recognition performance (SNR: −6 and −2 dB) [58] and an increase in Speech Reception Threshold (SNR required to achieve a 50% intelligibility) [6] when increasing the number of competing talkers from one to four (two to four in [6]). However, firm conclusions cannot be established due to the discrepancies in the experimental task (speech perception for the adults, a more cognitively demanding one—comprehension—for the children) and the listening conditions (SNR).

Concerning the second research question (role of individual factors), a direct assessment of students’ executive functions and noise sensitivity made it possible to test whether these individual characteristics moderate the effect of multi-talker noise on the task. Selective attention was found to interact with the listening condition on both accuracy and response time. Students with higher selective attention had a lower performance (lower accuracy and longer RTs) in the two-talker condition, whereas students with low selective attention were slower in the 2T condition, but equally accurate when background noise was present. The latter finding can be explained by the idea that an increase in the masking potential of the interferes leads to the deployment of more processing effort and, hence, longer response times as predicted by the ELU model [33]. The former finding was not expected. It might be hypothesized that students with higher selective attention were not only able to segregate the two streams of information (target and noise) but, due to the favorable acoustical conditions, they could also release some spare cognitive capacity to attend the to-be-neglected stream. Said differently, in the 2T case, they were able to perform two comprehension tasks: one for the target (with accuracy still close to ceiling) and one for the masker, whose accuracy is unknown. This explanation is consistent with the data in the 4T condition, in which the same group could not maintain both streams and, hence, focused only on the target, achieving slightly better accuracy and a decrease in RT. The hypothesis is consistent with the finding that, up to two talkers, listeners are able to correctly recognize the number of speakers and to process—at least to a certain extent—all the speech streams [59]. For three or more talkers, when the single streams might no longer be discernible, the background voices would be processed as a whole, yielding a lower impact on the students’ performance. 

The finding that selective attention, but not working memory, interacted with the effect of the listening condition is in line with results from adults [60] suggesting that the ability to inhibit information rather than working memory is the key factor in mediating the effect of noise in a reading comprehension task. The result was replicated for children when the effect of noise on a creative task was explored [61]. Similarly, in [35], a significant association was found between a measure of auditory attention and children’s speech perception in a noise plus reverberation condition, but not in noise alone, suggesting that attention skills might be specifically related to the ability to use the temporal cues in the speech signal (which are mainly degraded by the presence of reverberation).

The subjective measure of listening effort was instead moderated by the self-rated noise sensitivity of the students, with sensitive children perceiving increased effort with two interfering speakers compared to the other two conditions, and non-sensitive children perceiving more effort in noisy compared to quiet conditions. Therefore, it appears that noise-sensitive children are not more vulnerable to the effects of noise as a group per se, but they are more at risk depending on the specific characteristics of the background noise, namely the amount of informational masking. A role for the amount of informational masking in the relationship between task performance and students’ noise sensitivity was also found in [39], even though an opposite pattern was observed (high-sensitivity students: no difference between noisy conditions; low-sensitivity students: lower performance in a writing task in three- compared to 14-talker). Differences in the task (writing vs. comprehension), task modality (visual vs. auditory), and participants’ age (university students vs. primary-school students) might be responsible for the change in the pattern of association, but further dedicated studies are needed to directly explore the discrepancy. Moreover, our results show that the pattern of association between individual characteristics and listening effort depends on the specific measure of effort. Even though literature exploring this association is still scarce, a similar finding was observed in two recent studies, with adults [62] and children [41]. 

The third research question dealt with the role of the classroom sound environment on outcomes of confidence and motivation. We found that students’ motivation was modulated by their cognitive abilities and, in particular, by working memory capacity. Differences were mainly observed between quiet and the two-competing-speakers condition. In particular, students with low working memory capacity were less motivated to perform the listening task in noise than in quiet. Conversely, students with high working memory capacity indicated more motivation in noise compared to quiet. It might be hypothesized that the latter result could be related to an increase in the level of arousal provided by the presence of noise [63], influencing interest and attention to the task and, consequently, motivation to complete it. However, as our experimental task lasted less than an hour, it would be of interest to test whether the same result could be replicated for longer exposure to background noise or task duration. As pointed out in [19], whereas the negative effect of noise on cognitive functioning in children is well-established, research on motivation in relation to noise, and on the possible relation between the cognitive effects of noise and motivation, is still limited. Further research could indeed provide information to better understand the effects of the sound environment on students’ learning and well-being in classrooms.

The sound environment might also influence a listener’s level of confidence in her/his capacity in doing the task. Our results indicate that the listening condition does not have a direct effect on the student’s confidence, but it is moderated by the individual noise sensitivity. In particular, high-sensitivity students rated lower confidence in their capacity of doing the task in the two-talker condition than in quiet. No differences were found for students with low noise sensitivity. As no difference in task accuracy was observed between the two groups of students in these listening conditions, it might be speculated that low-sensitivity students had a lower metacognitive confidence: a lower ability to reason about the actual impact of noise on the comprehension of the message and, in turn, take actions to cope with the problem. Conversely, if we are to create a comfortable classroom environment, to be used as an educational resource [64], students should become aware of the impact of noise on the task at hand; this promotes their capacity to react to the noisy environment and interact with it, intervening autonomously to adapt it to their own needs (e.g., reducing the sound level, asking the teacher to repeat the sentence). In this sense, the appropriate acoustic design of learning spaces (that already prompts a reduction in the sound levels related to the student activity [65]) should be combined with interventions aimed at increasing teachers’ and students’ awareness [38]. The combined effect would be particularly beneficial for the most noise-sensitive students.

This study has limitations that could be addressed in future studies. First, we limited our investigation to cognitive abilities and did not explore the effect of linguistic skills as moderators of the listening condition. A previous study, on listening comprehension for students of the same age range, indicated that baseline literacy skills do not moderate the effect of background noise (traffic, classroom noise) and instead provide an overall advantage, whatever the listening conditions [66]. Future studies should explore whether the same result holds true in the case of a background noise of competing talkers. A second limitation pertains to our choice of working memory task (2-back task). This task requires high levels of selective and sustained attention and could, thus, measure more executive function components than the working-memory subcomponent alone [37]. More focused measures of verbal or visual-spatial working memory could provide better insight into the role of WM in dealing with the presence of informative background noise. Finally, we presented the children with a listening task. It might be hypothesized that different patterns of association between task performance and individual characteristics would be observed in the case of an academic task presented in the visual domain (e.g., a reading task). More research is needed to extend the present findings to different academic tasks and presentation modalities.

## 5. Conclusions

This study examined the effects of multiple competing speakers and individual characteristics (cognitive abilities, noise sensitivity) on 10- to 13-year-old students’ accuracy and effort in a listening comprehension task. The effects on the subjective dimensions of motivation and confidence in doing the task were examined as well. The results indicate no direct effect of the number of competing speakers on the study outcomes. However, the effect of the listening conditions was moderated by the individual characteristics: selective attention for accuracy and response time, noise sensitivity for perceived effort and confidence, and working memory for motivation. When the effect of the individual characteristics was included in the analysis, the background condition with two competing talkers appeared to have mostly negative effects on the students at greater risk (e.g., with higher noise sensitivity or lower working memory), owing to its greater informational masking. However, it was also found that children with higher selective attention had slightly lower accuracy and longer response time in the same two-talkers condition. A possible explanation for this last finding was their ability to attend both streams, instead of neglecting the maskers, which was not the case for the four-talker condition.

## Data Availability

The data presented in this study are available on request from the corresponding author. The data are not publicly available due to participants being informed that only group scores would be made public.

## References

[1] Vander Ghinst M., Bourguignon M., Niesen M., Wens V., Hassid S., Choufani G., Jousmäki V., Hari R., Goldman S., De Tiège X. (2019). Cortical tracking of speech-in-noise develops from childhood to adulthood. J. Neurosci..

[2] Leibold L.J. (2017). Speech perception in complex acoustic environments: Developmental effects. J. Speech Lang. Hear. Res..

[3] Mattys S.L., Davis M.H., Bradlow A.R., Scott S.K. (2012). Speech recognition in adverse conditions: A review. Lang. Cogn..

[4] Cooke M. (2006). A glimpsing model of speech perception in noise. J. Acoust. Soc. Am..

[5] Freyman R.L., Balakrishnan U., Helfer K.S. (2004). Effect of number of masking talkers and auditory priming on informational masking in speech recognition. J. Acoust. Soc. Am..

[6] Culling J.F. (2016). Speech intelligibility in virtual restaurants. J. Acoust. Soc. Am..

[7] Leibold L.J., Buss E. (2019). Masked speech recognition in school-age children. Front. Psychol..

[8] Corbin N.E., Bonino A.Y., Buss E., Leibold L.J. (2016). Development of open-set word recognition in children: Speech-shaped noise and two-talker speech maskers. Ear Hear..

[9] Peng Z.E., Pausch F., Fels J. (2021). Spatial release from masking in reverberation for school-age children. J. Acoust. Soc. Am..

[10] von Lochow H., Lyberg-Åhlander V., Sahlén B., Kastberg T., Brännström K.J. (2018). The effect of voice quality and competing speakers in a passage comprehension task: Performance in relation to cognitive functioning in children with normal hearing. Logoped. Phoniatr. Vocol..

[11] von Lochow H., Lyberg-Åhland V., Sahlén B., Kastberg T., Brännström K.J. (2018). The effect of voice quality and competing speakers in a passage comprehension task: Perceived effort in relation to cognitive functioning and performance in children with normal hearing. Logoped. Phoniatr. Vocol..

[12] Pichora-Fuller M.K., Kramer S.E., Eckert M.A., Edwards B., Hornsby B.W.Y., Humes L.E., Lemke U., Lunner T., Matthen M., Mackersie C.L. (2016). Hearing impairment and cognitive energy: The framework for understanding effortful listening (FUEL). Ear Hear..

[13] Prodi N., Visentin C. (2015). Listening efficiency during lessons under various types of noise. J. Acoust. Soc. Am..

[14] Prodi N., Visentin C. (2022). A slight increase in reverberation time in the classroom affects performance and behavioral listening effort. Ear Hear..

[15] Key A.P., Gustafson S.J., Rentmeester L., Hornsby B.W.Y., Bess F.H. (2017). Speech-processing fatigue in children: Auditory event-related potential and behavioral measures. J. Speech Lang. Hear. Res..

[16] Hétu R., Riverin L., Lalande N., Getty L., St-cyr C. (1988). Qualitative analysis of the handicap associated with occupational hearing loss. Br. J. Audiol..

[17] Maier S.F., Seligman M.E. (1976). Learned helplessness: Theory and evidence. J. Exp. Psychol. Gen..

[18] Evans G.W., Stecker R. (2004). Motivational consequences of environmental stress. J. Environ. Psychol..

[19] Dohmen M., Braat-Eggen E., Kemperman A., Hornikx M. (2022). The effects of noise on cognitive performance and helplessness in childhood: A review. Int. J. Environ. Res. Public Health.

[20] Lemke U., Besser J. (2016). Cognitive load and listening effort: Concepts and age-related considerations. Ear Hear..

[21] Peelle J.E. (2018). Listening effort: How the cognitive consequences of acoustic challenge are reflected in brain and behavior. Ear Hear..

[22] Lyberg-Åhlander V., Brännström K.J., Sahlén B.S. (2015). On the interaction of speakers’ voice quality, ambient noise and task complexity with children’s listening comprehension and cognition. Front. Psychol..

[23] Rudner M., Lyberg-Åhlander V., Brännström J., Nirme J., Pichora-Fuller M.K., Sahlén B. (2018). Listening comprehension and listening effort in the primary school classroom. Front. Psychol..

[24] Klatte M., Hellbrück J., Seidel J., Leistner P. (2010). Effects of classroom acoustics on performance and well-being in elementary school children: A field study. Environ. Behav..

[25] Lewinski P. (2015). Effects of classrooms’ architecture on academic performance in view of telic versus paratelic motivation: A review. Front. Psychol..

[26] Dienes Z. (2008). Subjective measures of unconscious knowledge. Prog. Brain Res..

[27] Carbon C.-C. (2020). Wearing face masks strongly confuses counterparts in reading emotions. Front. Psychol..

[28] Giovanelli E., Valzolgher C., Gessa E., Todeschini M., Pavani F. (2021). Unmasking the difficulty of listening to talkers with masks: Lessons from the COVID-19 pandemic. I-Perception.

[29] Dockrell J.E., Shield B.M. (2006). Acoustical barriers in classrooms: The impact of noise on performance in the classroom. Br. Educ. Res. J..

[30] Altomonte S., Allen J., Bluyssen P.M., Brager G., Heschong L., Loder A., Schiavon S., Veitch J.A., Wang L., Wargocki P. (2020). Ten questions concerning well-being in the built environment. Build. Environ..

[31] Reinten J., Braat-Eggen P.E., Hornikx M., Kort H.S.M., Kohlrausch A. (2017). The indoor sound environment and human task performance: A literature review on the role of room acoustics. Build. Environ.

[32] Astolfi A., Puglisi G.E., Murgia S., Minelli G., Pellerey F., Prato A., Sacco T. (2019). Influence of classroom acoustics on noise disturbance and well-being for first graders. Front. Psychol..

[33] Rönnberg J., Lunner T., Zekveld A., Sörqvist P., Danielsson H., Lyxell B., Dahlström Ö., Signoret C., Stenfelt S., Pichora-Fuller M.K. (2013). The ease of language understanding (ELU) model: Theoretical, empirical, and clinical advances. Front. Syst. Neurosci..

[34] Sullivan J.R., Osman H., Schafer E.C. (2015). The effect of noise on the relationship between auditory working memory and comprehension in school-age children. J. Speech Lang. Hear. Res..

[35] McCreery R.W., Walker E.A., Spratford M., Lewis D., Brennan M. (2019). Auditory, cognitive, and linguistic factors predict speech recognition in adverse listening conditions for children with hearing loss. Front. Neurosci..

[36] Klatte M., Bergström K., Lachmann T. (2013). Does noise affect learning? A short review on noise effects on cognitive performance in children. Front. Psychol..

[37] Diamond A. (2013). Executive Functions. Annu. Rev. Psychol..

[38] Massonnié J., Frasseto P., Ng-Knight T., Gilligan-Lee K., Kirkham N., Mareschal D. (2022). Children’s effortful control skills, but not their prosocial skills, relate to their reactions to classroom noise. Int. J. Environ. Res. Public Health.

[39] Braat-Eggen E., Reinten J., Hornikx M., Kohlrausch A. (2020). The influence of background speech on a writing task in an open-plan study environment. Build Environ..

[40] Braat-Eggen E., v.d. Poll M.K., Hornikx M., Kohlrausch A. (2019). Auditory distraction in open-plan study environments: Effects of background speech and reverberation time on a collaboration task. Appl. Acoust..

[41] Visentin C., Pellegatti M., Garraffa M., Di Domenico A., Prodi N. (2023). How individual characteristics mediate performance, effort, and motivation during listening comprehension in noisy classrooms. Sci. Rep..

[42] Prodi N., Visentin C., Peretti A., Griguolo J., Bartolucci G.B. (2019). Investigating listening effort in classrooms for 5- to 7-year-old children. Lang. Speech Hear. Serv. Sch..

[43] Visentin C., Prodi N. (2018). A matrixed speech-in-noise test to discriminate favorable listening conditions by means of intelligibility and response time results. J. Speech Lang. Hear. Res..

[44] Stoet G. (2010). PsyToolkit: A software package for programming psychological experiments using Linux. Behav. Res. Methods.

[45] Stoet G. (2017). PsyToolkit: A novel web-based method for running online questionnaires and reaction-time experiments. Teach. Psychol..

[46] Leys C., Ley C., Klein O., Bernard P., Licata L. (2013). Detecting outliers: Do not use standard deviation around the mean, use absolute deviation around the median. J. Exp. Soc. Psychol..

[47] Meule A. (2017). Reporting and interpreting working memory performance in N-Back tasks. Front. Psychol..

[48] Senese V.P., Ruotolo F., Ruggiero G., Iachini T. (2012). The Italian version of the Weinstein noise sensitivity scale: Measurement invariance across age, sex, and context. Eur. J. Psychol. Assess..

[49] Cecchetto C. (2012). Comprendo: Batteria per la Comprensione di Frasi Negli Adulti.

[50] McGarrigle R., Rakusen L., Mattys S. (2021). Effortful listening under the microscope: Examining relations between pupillometric and subjective markers of effort and tiredness from listening. Psychophysiology.

[51] Lidestam B., Beskow J. (2006). Motivation and appraisal in perception of poorly specified speech. Scand. J. Psychol..

[52] Chan K.M.K., Li C., Ma E.P.M., Yiu E.M.L., McPherson B. (2015). Noise levels in an urban Asian school environment. Noise Health.

[53] Anwyl-Irvine A.L., Massonnié J., Flitton A., Kirkham N., Evershed J.K. (2020). Gorilla in our midst: An online behavioral experiment builder. Behav. Res..

[54] (2022). R: A Language and Environment for Statistical Computing [Computer Software]. https://www.r-project.org/.

[55] (2022). RStudio: Integrated Development Environment for R [Computer Software]. https://posit.co/products/open-source/rstudio/.

[56] Bates D., Mächler M., Bolker B., Walker S. (2015). Fitting linear mixed-effects models using lme4. J. Stat. Soft..

[57] Singmann H., Bolker B., Westfall J., Aust F., Ben-Shachar M.S. Afex: Analysis of Factorial Experiments. R Package (2022, Version 1.2-0). https://CRAN.R-project.org/package=afex.

[58] Rosen S., Souza P., Ekelund C., Majeed A.A. (2013). Listening to speech in a background of other talkers: Effects of talker number and noise vocoding. J. Acoust. Soc. Am..

[59] Kashino M., Hirahara T. (1996). One, Two, Many—Judging the Number of Concurrent Talkers. J. Acoust. Soc. Am..

[60] Sorqvist P., Halin N., Hygge S. (2010). Individual differences in susceptibility to the effects of speech on reading comprehension. Appl. Cognit. Psychol..

[61] Massonnié J., Rogers C.J., Mareschal D., Kirkham N.Z. (2019). Is classroom noise always bad for children? The contribution of age and selective attention to creative performance in noise. Front. Psychol..

[62] Francis A.L., Bent T., Schumaker J., Love J., Silbert N. (2021). Listener characteristics differentially affect self-reported and physiological measures of effort associated with two challenging listening conditions. Atten. Percept. Psychophys..

[63] Szalma J.L., Hancock P.A. (2011). Noise effects on human performance: A meta-analytic synthesis. Psychol. Bull..

[64] Laurìa A., Secchi S., Vessella L. (2020). Acoustic comfort as a salutogenic resource in learning environments—A proposal for the design of a system to improve the acoustic quality of classrooms. Sustainability.

[65] De Salvio D., D’Orazio D. (2022). Effectiveness of acoustic treatments and PA redesign by means of student activity and speech levels. Appl. Acoust..

[66] Prodi N., Visentin C., Borella E., Mammarella I.C., Di Domenico A. (2021). Using speech comprehension to qualify communication in classrooms: Influence of listening condition, task complexity and students’ age and linguistic abilities. Appl. Acoust..

